# A Hybrid Deep Learning and Improved SVM Framework for Real-Time Railroad Construction Personnel Detection with Multi-Scale Feature Optimization

**DOI:** 10.3390/s25072061

**Published:** 2025-03-26

**Authors:** Jianqiu Chen, Huan Xiong, Shixuan Zhou, Xiang Wang, Benxiao Lou, Longtang Ning, Qingwei Hu, Yang Tang, Guobin Gu

**Affiliations:** 1 Guangxi Key Laboratory of International Join for China-ASEAN Comprehensive Transportation, Nanning University, Nanning 530200, China; chenjianqiu@unn.edu.cn (J.C.); 13729375070@163.com (S.Z.); ninglongtang@unn.edu.cn (L.N.); kbb2020126@163.com (Y.T.); 2 Guangxi Key Laboratory of Intelligent Transportation System (ITS), Guilin University of Electronic Technology, Guilin 541004, China; xionghuan0695@163.com (H.X.); 17339200188@163.com (X.W.); 199711@163.com (Q.H.); 3 School of Traffic and Transportation Engineering, Central South University, Changsha 410075, China; benxiaolou@csu.edu.cn; 4 Faculty of Logistics and Digital Supply Chain, Naresuan University, Phitsanulok 65000, Thailand

**Keywords:** image recognition, personnel detection, ISVM, deep learning, image preprocessing, PCA

## Abstract

Railroad construction sites are high-risk environments where monitoring personnel safety is critical for preventing accidents and enhancing construction efficiency. Traditional manual monitoring and image processing methods exhibit deficiencies in real-time performance and accuracy. This paper proposes a railway worker detection method based on improved support vector machines (ISVM), while using non-local mean noise reduction and histogram equalisation pre-processing techniques to optimise image quality to improve detection efficiency and accuracy. Multiscale features are then extracted with Inception v3 and combined with principal component analysis (PCA) for dimensionality reduction. Finally, an SVM classification algorithm is employed for personnel detection. To process small sample categories, data enhancement techniques (e.g., random flip and rotation) and K-fold cross-validation are applied to optimize the model parameters. The experimental results demonstrate that the ISVM method significantly improves accuracy and real-time performance compared to traditional detection methods and single deep learning models. This method provides technical support for railroad construction safety monitoring and effectively addresses personnel detection tasks in complex construction environments.

## 1. Introduction

At intercity railway construction sites, heavy machinery such as large excavators and loaders shuttle back and forth, and a slight mistake could result in a collision with construction personnel. At the same time, operations such as intercity railway track laying require extremely high precision, and any deviation could pose a safety hazard [1]. Construction personnel are unevenly distributed, and operations are also carried out in some remote or narrow areas, which makes unified management and protection difficult. Moreover, there are many blind spots in monitoring at construction sites. Traditional manual monitoring methods are limited by manpower and the field of view, making it difficult to detect potential hazards in these areas. Simple image processing methods also cannot accurately, and in real time, monitor the status of personnel and construction conditions due to factors such as changes in light and obstructions, making construction sites constantly at risk of accidents and seriously threatening the safety of construction personnel [2,3]. During railway construction, status monitoring and the real-time detection of construction personnel is of great significance for preventing accidents and improving construction efficiency. However, due to the complex construction site environment, uneven distribution of personnel, blind spots in monitoring, and other challenges, it is difficult for traditional manual monitoring and simple image processing methods to meet the need for real-time and accurate information.

In recent years, deep learning technologies have led to breakthroughs in image recognition methods based on convolutional neural networks (CNNs), offering new possibilities for target detection in complex environments [4,5]. However, the application of deep learning methods requires large amounts of labelled data and faces limitations in real-time performance, particularly under hardware resource constraints. In contrast, traditional machine learning methods exhibit better generalization performance and lower computational complexity in small sample environments.

To address these challenges, this study proposes a railroad construction personnel detection method based on an improved support vector machine (ISVM), which combines the feature extraction capability of deep learning with the classification capability of traditional machine learning to enhance detection efficiency and accuracy. This study classifies complex construction environments and employs image preprocessing techniques, such as non-local mean (NLM) noise reduction and histogram equalization, to improve image detail retention and contrast, thus laying a solid foundation for subsequent feature extraction and classification. A personnel detection framework, designed to achieve both accuracy and real-time performance, extracts multi-scale features using the deep learning model Inception v3, removes redundant features through principal component analysis (PCA) dimensionality reduction, and combines them with the efficient support vector machine (SVM) classification algorithm. Additionally, to address the challenge of small-sample category detection, this study introduces data enhancement techniques, such as random flip and rotation, to increase sample diversity, and optimizes model parameters through K-fold cross-validation, further enhancing the robustness and generalization ability of the algorithm.

This study combines the feature extraction advantages of deep learning with the efficient classification features of traditional machine learning to design a comprehensive detection process for the complexity of railroad construction environments. The experimental results demonstrate that the method outperforms traditional detection methods and individual deep learning models in both accuracy and real-time performance, providing technical support for railroad construction safety monitoring.

## 2. Literature Review

Image recognition is a core task in the field of computer vision, with its research dating back to the 1960s. With advancements in computing power and the widespread application of deep learning, image recognition algorithms have evolved from traditional methods to modern deep learning techniques. Traditional image recognition algorithms depend on manual feature extraction techniques, such as the scale-invariant feature transform (SIFT), speeded-up robust feature (SURF), and histogram of gradient directions (HOG) methods [6,7,8,9], which classify images based on local features. However, these methods typically perform poorly in complex contexts and require extensive manual design.

In recent years, convolutional neural networks (CNNs), as a deep learning method, have become a mainstream technique for image recognition [10,11]. AlexNet [12] overcomes the limitations of traditional methods by introducing deep convolutional networks, achieving remarkable success in the ImageNet Large-Scale Visual Recognition Challenge and marking the successful application of deep learning in computer vision. Zhang [13] proposed a multi-scale visual attribute co-attention (mVACA) model that enhances zero-shot image recognition accuracy by combining attribute-related attention with visual self-attention, achieving competitive performance on several benchmark tasks. Since then, deep network models, such as VGG [14] and ResNet [15], have been proposed to further improve image classification accuracy. Although deep learning models have achieved significant breakthroughs in recognition accuracy, their demand for large-scale labelled data and computational resources remains a considerable challenge [16]. Additionally, target detection methods, such as Faster R-CNN [17] and YOLO [18], have also been widely applied in image recognition. Faster R-CNN improves target detection speed and accuracy through a region-generating network (RPN), while YOLO adopts an end-to-end training method to enhance real-time detection capability. However, YOLO’s performance in complex backgrounds and small object detection still has limitations. Additionally, methods based on the transformer architecture [19], such as DETR (detection transformer) [20], have been applied in image recognition research. DETR is better at handling long-distance dependencies through its self-attention mechanism and transformer structure, making it suitable for complex detection tasks. However, its computational complexity is relatively high, especially when trained on large-scale datasets, requiring more computational resources.

With the rapid development of artificial intelligence and deep learning technologies, automated monitoring systems based on image recognition have emerged as an effective solution to the limitations of traditional manual detection. Image recognition technology has been widely applied in various aspects of railroad construction, equipment monitoring, and track monitoring. In an experiment applying multimodal learning to detect hazardous actions using RGB-D inputs, Amel [21] evaluated various fusion strategies and modal encoders to determine the most effective way to capture complex cross-modal interactions, finding that the MultConcat multimodal fusion method achieves excellent results in construction safety detection in railroad construction. Ying [22] adopted an optimized computer recognition system and algorithmic learning method for image recognition in construction, effectively improving classification accuracy through training and testing, thus enhancing the automation and precision of the safety monitoring system at the construction site. Yang O [23] proposed an indoor moving target recognition and tracking method based on the collaborative information fusion of RFID and charge-coupled devices (CCDs) to track the location of construction workers in real time and ensure safety. Cao [24] proposed a railroad intrusion detection method, RailDet, using dynamic intrusion regions and a lightweight neural network for railroad construction intrusion detection. The method first utilizes an intrusion localization algorithm to obtain dynamic intrusion regions and then applies lightweight neural network-based target recognition to process these regions and identify railroad intrusions. Jerry [25] proposed a real-time train monitoring system based on the ZigBee/IEEE 802.15.4 protocol, enabling the monitoring, control, and performance evaluation of running fixed-route trains with low cost and low power consumption. Zhang [26] proposed a dynamic neural network detection model for rockfall intrusion detection in railroad slope construction, initially relying on the YOLOv5 neural network. The model uses an activation function suitable for the target scenario, solving the overfitting problem to achieve accurate target recognition. It dynamically detects rolling rocks by integrating a background subtraction algorithm based on Gaussian mixture models and uses monocular vision techniques to capture target dimensions, broadening detection information.

In railroad transportation, monitoring and controlling equipment is crucial to ensuring the safe and efficient operation of railroads. The operational status of railroad equipment, such as signalling systems, railroad vehicles, and their ancillary facilities, is critical to railroad safety. Image recognition technology enables the real-time monitoring of equipment and timely detection of failures or abnormalities through efficient visual analysis methods. To meet the demand for fast and accurate automatic inspection of railroad tunnel equipment maintenance, Duan Y et al. [27] proposed an automatic inspection solution based on panoramic imaging and object recognition. They installed a hyperbolic folding and reflecting panoramic imaging system on the inspection vehicle to capture a wide field of view while shielding highly dynamic phenomena at the tunnel exit. Additionally, they proposed a YOLOv5-CCFE-based railroad equipment identification model that meets the requirements for automatic inspection along the railroad tunnel. Aiming to detect the production of wheel pairs with challenges such as tilted characters and fixed positions affecting detection, Xu [28] proposed a deep learning-based method. First, a tilted character detection network was constructed using Faster R-CNN. Second, a tilted character correction network was designed to categorize and correct the direction of flipped characters. Finally, a character recognition network based on the convolutional recurrent neural network (CRNN) was constructed to recognize wheel-to-wheel characters. To address the low efficiency and accuracy of character recognition and condition monitoring in equipment detection, Wang [29] improved detection and data analysis methods by optimizing the genetic algorithm and the support vector machine (SVM) classifier, thus enhancing the accuracy and real-time performance of substation equipment condition monitoring. Additionally, in wheel defect detection, Gibert [30] proposed using artificial neural network technology to identify defects in ultrasound B-scan images by applying a noise reduction filtering algorithm and a feature extraction algorithm. Two back-propagation neural networks with hidden layers were constructed, one to identify noise and the other to identify echoes of real defects, ultimately achieving a high recognition rate. In railroad fastener detection research, Aydin [31] proposed a method to classify defects in rail fasteners by determining the locations of rails and sleepers based on high-contrast images. Features were extracted from two fully connected layers in an improved lightweight convolutional neural network (LCNN) model, and feature vectors were constructed to classify defects in fasteners. In drip detection research in railroad contact networks, Tan [32] proposed a multi-algorithm fusion image processing technique to construct a drip identification and failure defect-detection model based on deep learning algorithms and a sub-pixel level drip defect-detection model. This method achieves high-precision detection of drip failures and defects and utilizes a Faster R-CNN-based detection model for identifying the localization of dripping and for the failure detection of bent slack and fractured droppers. In hanging chord detection research in railroad contact networks, Zhang [33] proposed a YOLOv5-based target detection algorithm for hanging chord defects, using the MobileNetv3 module as a highly efficient and lightweight backbone feature extraction network. Depth-separable convolution was used instead of standard convolution, and the BiFPN feature pyramid structure was introduced to fuse different feature layers in the necking network, improving detection accuracy. In the online condition monitoring of pantograph slide plates, Wei [34] proposed an intelligent method based on deep learning and image processing techniques. In the first stage, a pantograph defect detection neural network (PDDNet) is used for defect detection and identification. In the second stage, five key criteria for assessing wear conditions are proposed, and wear edge estimation based on image processing techniques is investigated in detail.

Track monitoring is a critical component in maintaining a safe and reliable railroad system. Traditional manual track inspection methods are labour intensive and prone to errors. Image recognition techniques offer a more efficient and accurate method for detecting track defects such as cracks, wear, and misalignment. Railroad lines are among the most erosion-prone structures. Nogueira [35] proposed a novel deep learning-based framework for identifying and monitoring erosion on railroad lines, finding through evaluation that the dynamic expansion convolutional network provides the best recognition, and integrating it into the framework for effective erosion recognition. To reduce accidents caused by collisions with obstacles on tracks during train operations, Felipe [36] addressed the problem of identifying broken rails on a double-track railroad line by using principal component analysis (PCA) to classify current data, detect and locate track damage, optimize maintenance tasks, and ensure track integrity. Guo [37] proposed a new dual-magnet design (DMPS-EMAT) that improves signal strength by optimizing the positions of the magnets and coils, making long-range monitoring of tracks more effective. Wang [38] proposed a novel resonant triple-coil sensor to sense thermoacoustic signals for detecting internal defects in rails. A polynomial classifier trained with a support vector machine algorithm was used for defect recognition, and experiments demonstrated that the system achieved 96.4% accuracy, enabling intelligent defect detection in rails. For detecting and identifying rail geometry, Cheng [39] proposed a new line recognition algorithm (NLRA), which effectively filters out noise using partial differential equations for data preprocessing. The algorithm utilizes curve design parameters and combines noise-resistant line identification with multi-segment line-fitting technology to accurately identify each rail segment. Yue [40] proposed an automatic identification algorithm for detecting hidden defects behind railroad tunnel linings, using a panoramic driving perception network (YOLOP) model to estimate the lining thickness in GPR B-scan images of tunnels. The model employs a real GPR dataset of underground objects for migration learning, creating a dataset of real GPR B-scan images of tunnel linings, and is retrained and evaluated. In railroad surface defect detection, Min [41] proposed a lightweight two-stage architecture, including the railway crop network (RC-Net) and defect removal variational auto-encoder (DR-VAE), which detects defects through self-supervised learning. The system reduces random reconstruction errors by introducing a distribution capacity attenuation factor and uses the residuals of original and reconstructed images to segment defects, effectively addressing issues of insufficient defect samples and an imbalance of positive and negative samples. Zhuang [42] addressed the problem of vibration signal analysis in track monitoring by adopting a deep learning framework combined with an integration strategy of heterogeneous factors. The approach solved issues of insufficient databases and feature extraction difficulty through data-driven methods, achieving highly accurate track defect detection and severity prediction. Kim [43] developed a segmentation model by modifying the full convolutional network model from a deep learning perspective, normalizing the variance and mean of the data images. The model was trained using these images to segment defective regions for the automatic detection and segmentation of defects on the railroad surface. In a study addressing small or broken cracks in the track, Rampriya [44] proposed a fusion model using deep convolutional neural networks for fault detection and the semantic segmentation of the railroad line. The ResNet50 network is used for fault identification, and the Deep Residual U-Net network is used for semantic segmentation. The identified defects are isolated, and decisions on whether to check or ignore detected faults are made based on the generated binary image segments.

Current methods struggle to balance accuracy and efficiency: deep learning demands excessive data and resources, while traditional algorithms lack robustness in complex environments. Hybrid approaches often fail to address real-time multi-scale detection in remote or cluttered construction areas with uneven personnel distribution. Moreover, most solutions focus on isolated tasks rather than offering a unified framework adaptable to dynamic safety monitoring, environmental challenges, and hardware constraints in intercity railway construction. Overall, the application of image recognition technology in the railroad industry not only enhances the efficiency and accuracy of monitoring but also fosters the development of automation and intelligence [45,46,47], with the literature summarized in Table 1.

Therefore, although deep learning methods currently dominate the field of image classification, with their end-to-end feature learning and transfer learning capabilities showing significant advantages in most scenarios, the theoretical properties and engineering value of traditional classifiers still merit in-depth exploration. Compared to traditional algorithms that rely on strong feature engineering assumptions or are limited by computational complexity, support vector machines (SVMs) have achieved a unique balance between small sample learning and model reliability through structural risk minimisation theory and kernel space mapping mechanisms. Their maximum interval classification strategy theoretically guarantees robustness to sample perturbations, while the kernel function’s implicit high-dimensional mapping of nonlinear problems breaks through the limitations of traditional linear models in complex pattern recognition. Although deep learning has significant advantages at the level of feature abstraction, the SVM continues to play a key role in areas such as industrial inspection and financial security, where the mathematical interpretability of the decision process and the deployment efficiency of lightweight architectures require strict algorithmic transparency.

## 3. Image Processing and Algorithm Design

### 3.1. Image Collection and Processing

The current traditional methods that are mainly relied on for the safety monitoring of railway construction have obvious deficiencies. Although manual inspection and visual monitoring are flexible, they are affected by manpower and subjective judgment, cannot achieve real-time monitoring around the clock, and have a high missed inspection rate. Electronic tags and wearable devices, such as the RFID-CCD fusion solution, can track the location of workers, but they face high deployment costs, are easily disturbed by metal environments, and have a high false positioning rate, which limits their large-scale application. Basic image processing techniques based on OpenCV, such as inter-frame difference and optical flow, have acceptable computational efficiency, but have a high false detection rate in complex scenes.

**Figure 1:**
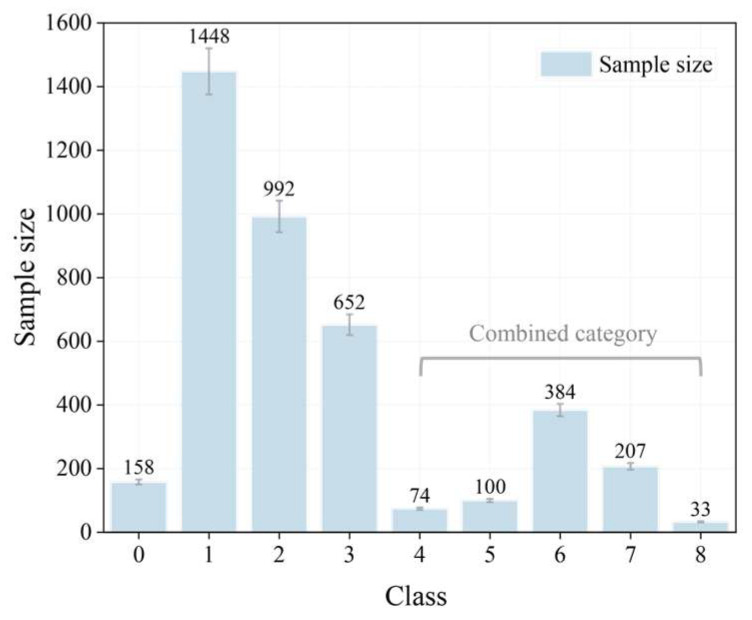
Sample distribution of detection tasks.

In view of the shortcomings of the above traditional methods, we collected 3222 images of railway construction in complex scenarios. The main detection object was people, and the image resolution was 1920 × 1080. The image dataset was collected by monitoring, and each frame was taken. This study focuses on intercity railway construction scenarios, mainly for ground construction environments. Due to the complexity of railway construction scenarios and the large number of blind spots in monitoring, this study adopts a classification method to study the application of classification methods in monitoring in different scenarios. The dataset mainly collects image data from 14 cameras, and the sample size of each category is unevenly distributed. In deep learning, when the categories are unbalanced, the algorithm has difficulty identifying features, which can easily lead to overfitting. Therefore, five categories are defined. As shown in Figure 1, the number of categories in this dataset is 0–8, representing the number of people in each scene in this dataset, from 0 to 8. However, due to the obvious differences in the sample size of each category, such as the small numbers in category 4 and category 8, the scattered images of categories 4–8 and the small number of samples can be grouped into one category. Therefore, this study divides the number of categories into 5 categories.

#### 3.1.1. Image Noise Reduction

Traditional denoising methods (e.g., mean filtering and Gaussian filtering) typically smooth pixel values within localized regions, which can lead to the blurring of image details. In the complex railroad construction environment, this study adopts non-local mean filtering for noise reduction and detail preservation, based on the core idea that similar pixels in an image may be distributed across the entire image, not just within local regions. To mitigate the effect of weak noise on model training, the image is converted to a greyscale map. For a pixel i in the image, its denoised value u(i) is determined by the Formula (1) calculation.

(1) $$u(i)=\frac{\sum_{j\in \Omega }w(i,j)\cdot v(i,j)}{\sum_{j\in \Omega }w(i,j)}$$

where u(i) is the value of pixel j in the original image. Ω is the search window, which indicates the range of pixels to be searched in the image. w(i,j) is the weight, which indicates the similarity of pixel i and pixel j, satisfying w(i,j)≥0 and $\sum_{j\in \Omega }w(i,j)=1$.

**Figure 2:**
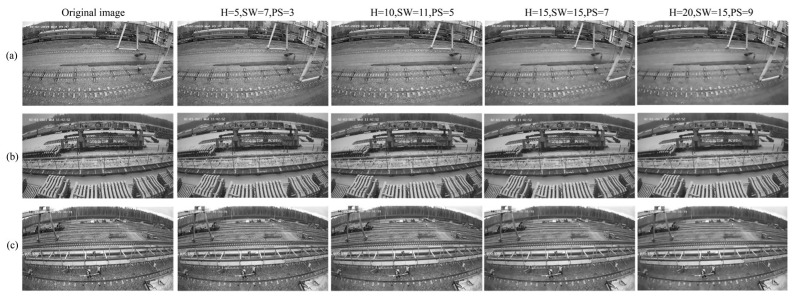
Effect of NLM noise reduction with different parameters. In scene (**a**), the noise decreases with the parameter increase, but the edges of the object tend to be blurred; in scene (**b**), the noise residue is obvious when the parameter is small, and the noise is effectively suppressed after the increase, but the texture details are overly smoothed; in scene (**c**), the parameter change improves the denoising effect and the image is smoother, but the higher parameter overprocesses the object contour and texture details. Overall, the three sets of scenarios visualize the balanced influence of non-local mean filtering parameters on the image denoising effect and feature retention.

The weight $w\left(i,j\right)$ is calculated as follows:

(2) $$w\left(i,j\right)=\exp\left(-\frac{{\left\|P\left(i\right)-P\left(j\right)\right\|}_{2}^{2}}{h^{2}}\right)$$

where P(i) and P(j) denote the image blocks (Patch) centred on pixels i and j, respectively. ${\left\|P(i)-P(j)\right\|}_{2}^{2}$ is the Euclidean distance between the two image blocks. H is the smoothing parameter that controls the denoising intensity. The larger the value of H, the smaller the weight difference, resulting in a stronger denoising effect, although it may lead to distortion. The effectiveness of non-local mean filtering depends on three key parameters: search window size, image block size, and the smoothing parameter. Figure 2 demonstrates the effect of non-local mean filtering on three scenes with varying parameters.

**Figure 3:**
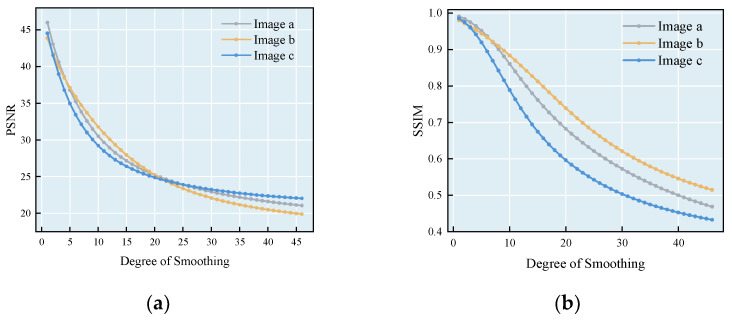
(**a**) PSNR and (**b**) SSIM sensitivity analysis.

Figure 3 shows the quantitative relationship between the peak signal-to-noise ratio (PSNR) and structural similarity index (SSIM) as a function of the smoothing parameter H under experimental conditions with a search window size of 15 × 15 pixels and an image block size of 9 × 9 pixels. The experimental results show that when the value of H increases from 1 to 45, PSNR and SSIM show a monotonous decreasing trend, which indicates that an excessively large smoothing parameter will lead to an exacerbation of the loss of image details. This study determined the optimal parameter H = 21 through experiments.

#### 3.1.2. Image Enhancement

This study uses global histogram equalisation (GHE) as the core enhancement method. It is worth noting that although GHE can improve the overall contrast, it can lead to two major problems: ① In scenes with high frequency details such as protective clothing and helmets, GHE will excessively enhance noise. ② In scenes with uneven light distribution, GHE’s global adjustment will destroy the natural light gradient, causing dark details to be lost.

To overcome the above problem, we used the GHE method to de-fog the image after NLM noise reduction. This suppresses most of the noise and avoids the problem of GHE over-enhancing the noise. For uneven light distribution, GHE’s adaptive enhancement of local sub-regions can still maintain the continuity of light and dark transitions in a greyscale space, so that key features such as the reflective helmet logo and dark areas can be preserved in a single channel. All of the images in this experiment have a greyscale range of [0, 255] and the number of grey levels L is 256, so let the number of pixels in the image with grey value $r_{k}$ be $n_{k}$ and the total number of pixels in the image be N. The probability distribution of the grey value $r_{k}$ is as follows:

(3) $$\begin{array}{cc} p\left(r_{k}\right)=\frac{n_{k}}{N}, & k=0,1,2,\ldots ,L-1 \end{array}$$

where $p\left(r_{k}\right)$ is the probability of grey value $n_{k}$, $r_{k}$ is the number of pixels with grey value N, and $T\left(r_{k}\right)$ is the total number of pixels in the image.

The cumulative distribution function F is defined as the sum of the probabilities of the grey value $r_{k}$ and the following grey values:

(4) $$\begin{array}{cc} T\left(r_{k}\right)=\sum_{j=0}^{k}p\left(r_{j}\right), & k=0,1,2,\ldots ,L-1 \end{array}$$

where $T\left(r_{k}\right)$ is the cumulative probability of the greyscale value.

Mapping the original greyscale value $r_{k}$ to the new greyscale value $s_{k}$, the mapping formula is as follows:

(5) $$s_{k}=\left(L-1\right)\cdot T\left(r_{k}\right)$$

where $s_{k}$ is the mapped grey value, and the range is still $\left[0,L-1\right]$. $T\left(r_{k}\right)$ is the normalized cumulative distribution function, and the value range is $\left[0,1\right]$.

Through the above mapping, the distribution of greyscale values in the original image is stretched or compressed, resulting in a more uniform histogram and enhanced image contrast.

**Figure 4:**
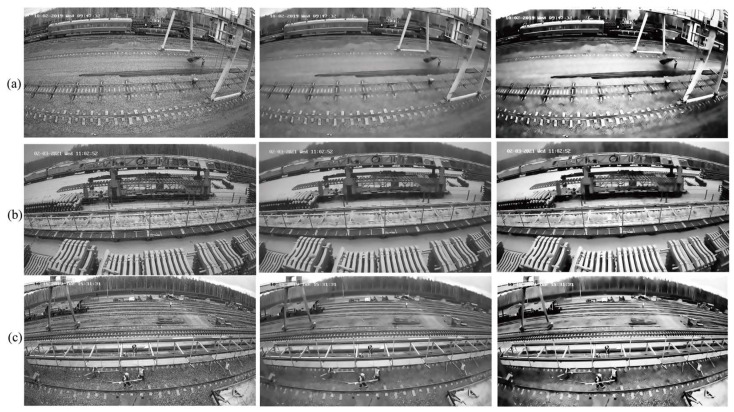
Comparison of details after enhancement of 3 groups of images (**a**) the original image (greyed out), (**b**) the image after NLM noise reduction, and (**c**) the en-hanced image after applying the global histogram equalization technique.

Figure 4 demonstrates the processing effect of three sets of images: the original image (greyed out), the image after NLM noise reduction, and the enhanced image after applying the global histogram equalization technique. By combining noise reduction and enhancement techniques, residual noise is significantly reduced, and the recognizability of the contour features of railroad construction workers is improved.

**Figure 5:**
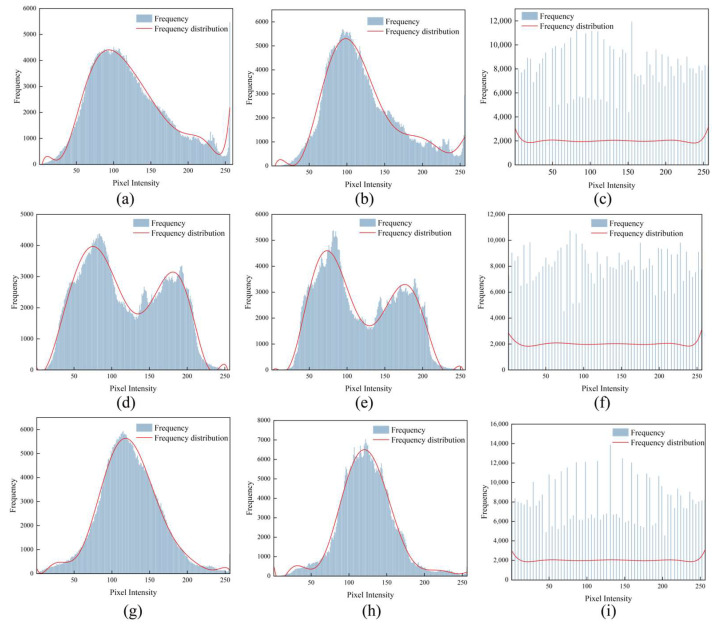
Frequency histogram of the number of pixels in 3 groups of images. The histograms of the original images (**a**,**d**,**g**) show different brightness distribu-tion characteristics. After NLM noise reduction (**b**,**e**,**h**), the histograms become smoother and the noise is significantly reduced. After applying global histogram equalization (**c**,**f**,**i**), the pixel intensity distribution tends to be uniform, enhancing the contrast and detail expression of the image.

Figure 5 shows the frequency histograms of the pixel intensity for three image sets, corresponding to the original image, the image after NLM noise reduction, and the image after global histogram equalisation. The histograms of the original images (a, d, and g) show different brightness distribution characteristics. After NLM noise reduction (b, e, and h), the histograms become smoother and the noise is significantly reduced. After applying global histogram equalization (c, f, and i), the pixel intensity distribution tends to be uniform, enhancing the contrast and detail expression of the image.

#### 3.1.3. Data Enhancement

In image enhancement strategies, common methods include operations such as rotation, flipping, occlusion, lighting changes, and scaling and cropping. Among these, rotation and flipping are lightweight transformations that avoid significant interference with the target structure by maintaining geometric invariance in the feature space. Their linear time complexity (O(N)) gives them a significant advantage in terms of computational efficiency, and they are particularly suited to scenarios with high real-time requirements. Occlusion enhancement can improve the robustness of the model to incomplete features by simulating scenes where parts of the target are missing. However, there is a risk of damaging the integrity of the features. Excessive occlusion (such as hard rectangular occlusion) can easily introduce artificial artefacts. The occlusion ratio must be strictly controlled to balance the enhancement effect and feature fidelity. Lighting variation enhancement can effectively improve model generalisation by adjusting parameters such as brightness and contrast to simulate complex lighting conditions. However, it has limited adaptability to scenes with complex textures, and improper parameter settings can distort image contrast. Parameter calibration is required in conjunction with scene characteristics.

**Figure 6:**
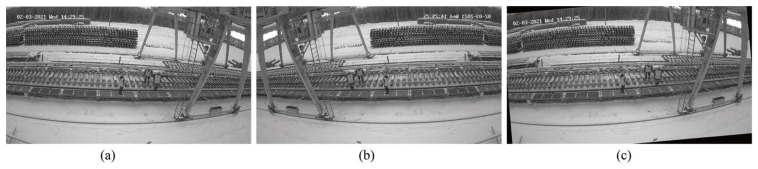
Data enhancement: (**a**) original image, (**b**) flipped image, and (**c**) rotated image (5°).

Due to the limited number of image samples (N ≤ 5) for certain scenes in this dataset, dividing the training, testing, and validation sets presents challenges. To increase the sample size for these scenes, as shown in Figure 6, random flip and rotation (5°) operations were performed on individual samples, and the original images were included in the validation set.

### 3.2. Algorithm Design

#### 3.2.1. Algorithm Design Ideas

Railroad construction personnel detection typically requires high real-time performance, but traditional feature extraction methods suffer from poor feature quality, high dimensionality, and long computation times, making it difficult to meet real-time construction requirements. Deep learning methods, such as convolutional neural networks (CNN), can extract high-quality features, but they require extensive training to achieve good classification results. In contrast, short-time feature extraction faces the challenges of poor feature quality and recognition performance.

In this study, we propose a railroad construction worker detection method, aiming to achieve the short-time extraction of high-quality features and improve detection efficiency and accuracy by optimizing deep learning pretraining and applying traditional machine learning methods. The feature extraction capabilities of deep learning and the classification abilities of traditional machine learning are fully utilized to achieve fast and accurate railroad construction personnel detection.

**Figure 7:**
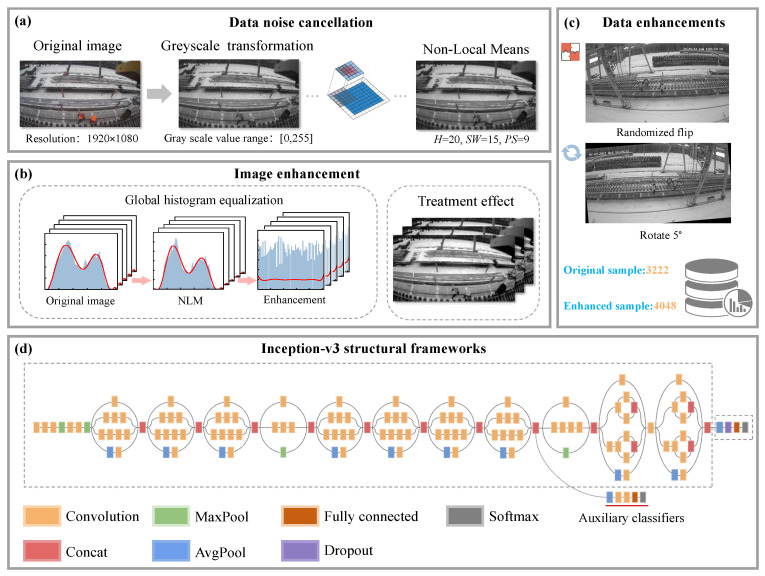
Schematic diagram of the technical flow of this experimental algorithm.

As shown in Figure 7, the algorithm design steps of this study are as follows:

Step 1: This experiment first converts the image to a greyscale image and then performs NLM denoising to reduce the noise in the image.

Step 2: The histogram equalization method is used to enhance the denoised image.

Step 3: For small batches of scene samples, the data samples are enhanced by random flipping and rotating by 5°. This reduces the problem of overfitting caused by the uneven category samples.

Step 4: Features are extracted using the Inception v3 network architecture. The final AvgPool layer is selected as the feature layer to extract multi-scale information.

Step 5: PCA is introduced into the AvgPool layer to reduce the risk of overfitting. Finally, the features are passed to an improved SVM for classification detection and recognition.

#### 3.2.2. Inception v3 Model

**Figure 8:**
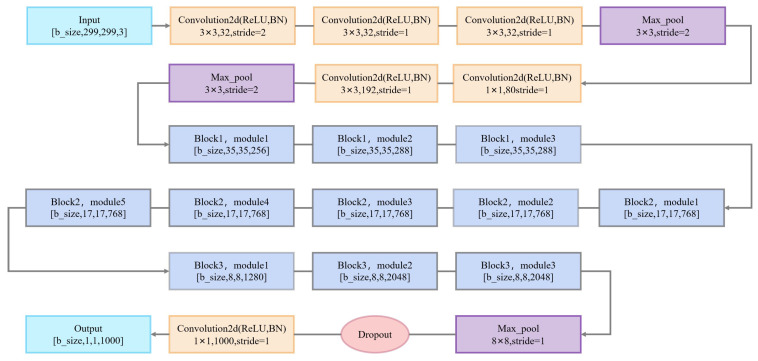
Inception v3 network architecture.

Inception v3 is a deep learning model based on an improved version of GoogLeNet. Inception v3 has achieved strong results in the ImageNet Large-Scale Visual Recognition Challenge (ILSVRC) and is widely used in image classification and object recognition tasks. As shown in Figure 8, the main feature of Inception v3 is the use of multiple parallel convolutional and pooling layers, along with auxiliary classifiers to improve model performance. This structure enables Inception v3 to efficiently learn features at different scales, enhancing the model’s generalization ability.

**Figure 9:**
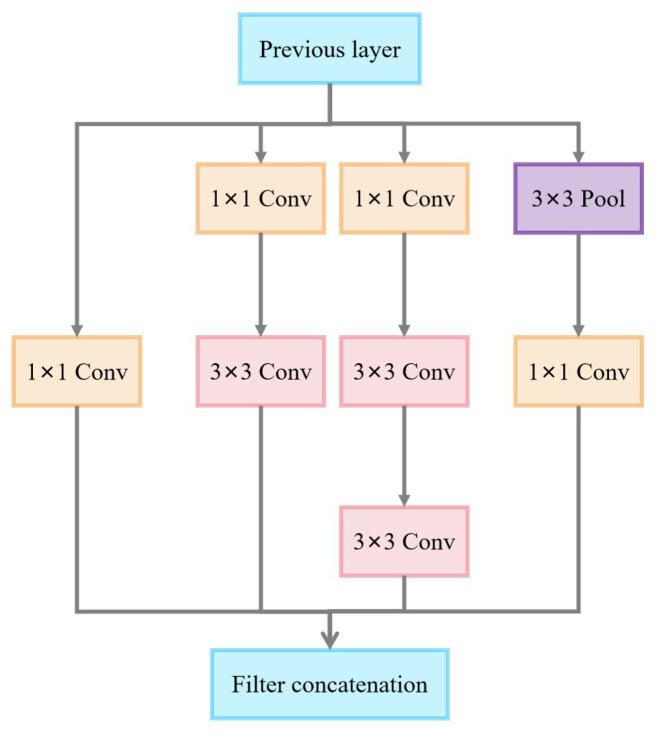
Inception v3-A module.

The Inception v3-A module employs a multiplexed parallel network structure with four parallel branches: a single 1 × 1 convolutional branch, a 1 × 1 convolutional branch connected to a 3 × 3 convolutional branch, a 1 × 1 convolutional branch connected to two 3 × 3 convolutional branches, and a 3 × 3 pooling branch connected to a 1 × 1 convolutional branch, as shown in Figure 9. Multi-scale feature extraction is achieved by combining convolution kernels of different scales, with 1 × 1 convolution used for dimensionality reduction to reduce computational costs. The parallel branching structure in the module not only increases the network’s width and enhances the model’s feature representation ability, but also enables the effective fusion of multi-scale features through the final feature concatenation operation.

**Figure 10:**
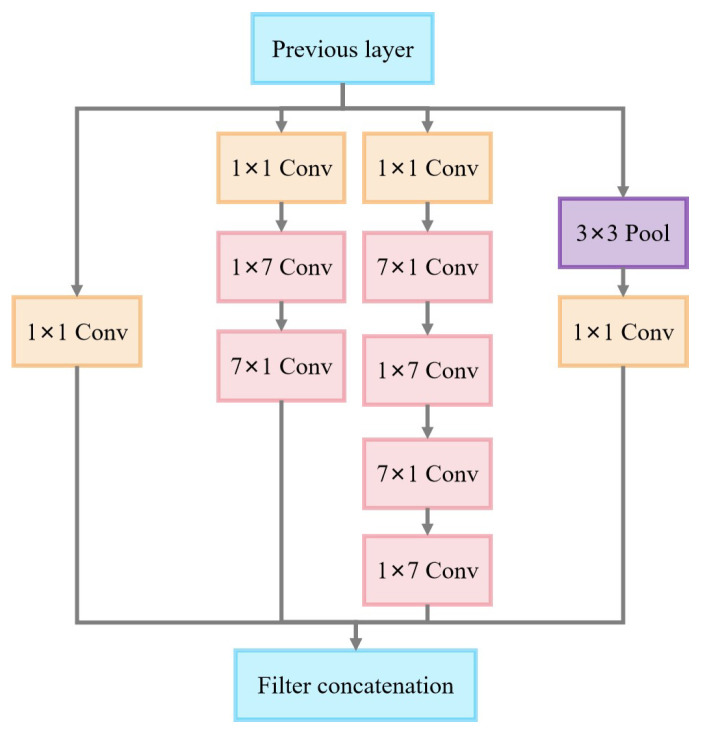
Inception v3-B module.

The core feature of the Inception-B module is the use of an asymmetric convolutional decomposition strategy. As shown in Figure 10, the module consists of four parallel paths, including a simple 1 × 1 convolutional channel, two depth channels based on asymmetric convolutional transforms, and a pooling channel. The two main branches in the module use a tandem combination of (1 × 7) and (7 × 1) convolutions, with the decomposition effectively replacing the functionality of traditional large-size convolutional kernels.

**Figure 11:**
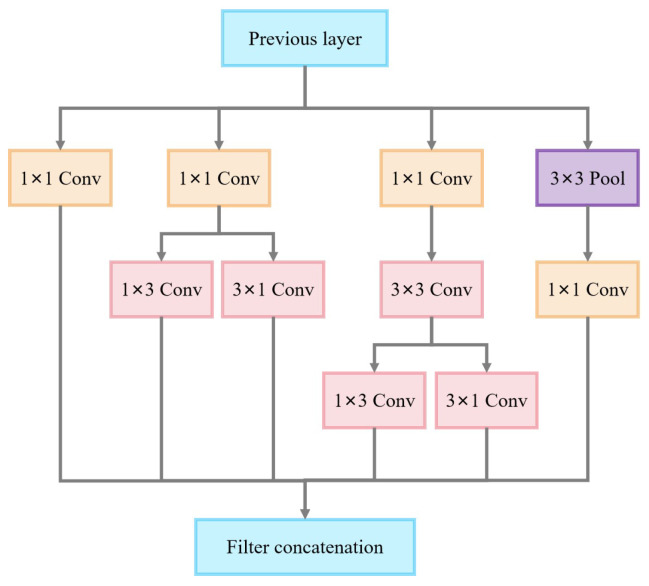
Inception v3-C module.

As shown in Figure 11, the widely used asymmetric convolutional design (with a kernel size of 3) in the Inception v3-C module strikes a balance between the model’s computational efficiency and feature extraction capabilities. Using the decomposition strategy, the module reduces the number of parameters and computational costs while ensuring the model captures a wider range of contextual information in the image. The feature maps fused through each branch synthesize information at different scales and abstraction levels.

**Figure 12:**
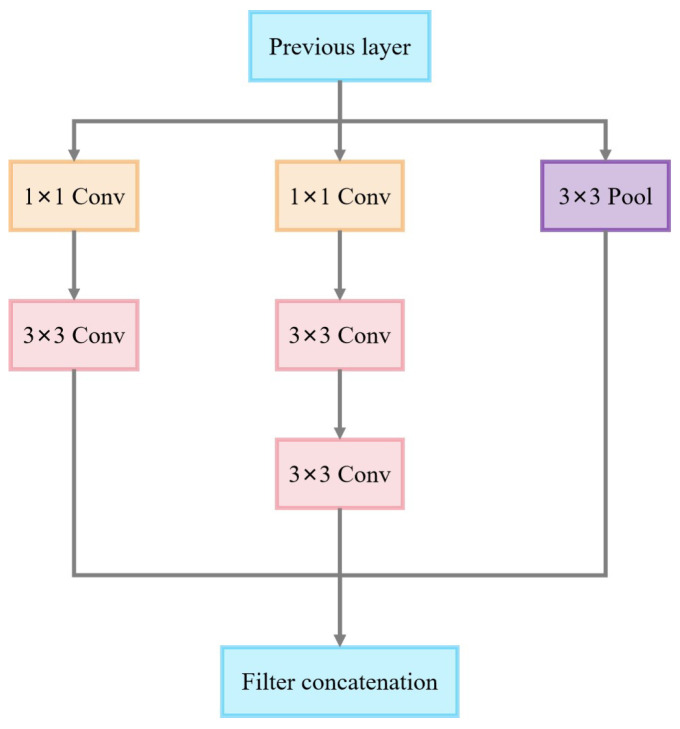
Inception v3-D module.

As shown in Figure 12, the Inception v3-D module features a multi-branch parallel architecture with three feature extraction paths optimized for dimensionality reduction via 1 × 1 convolution, thereby reducing computational complexity.

**Figure 13:**
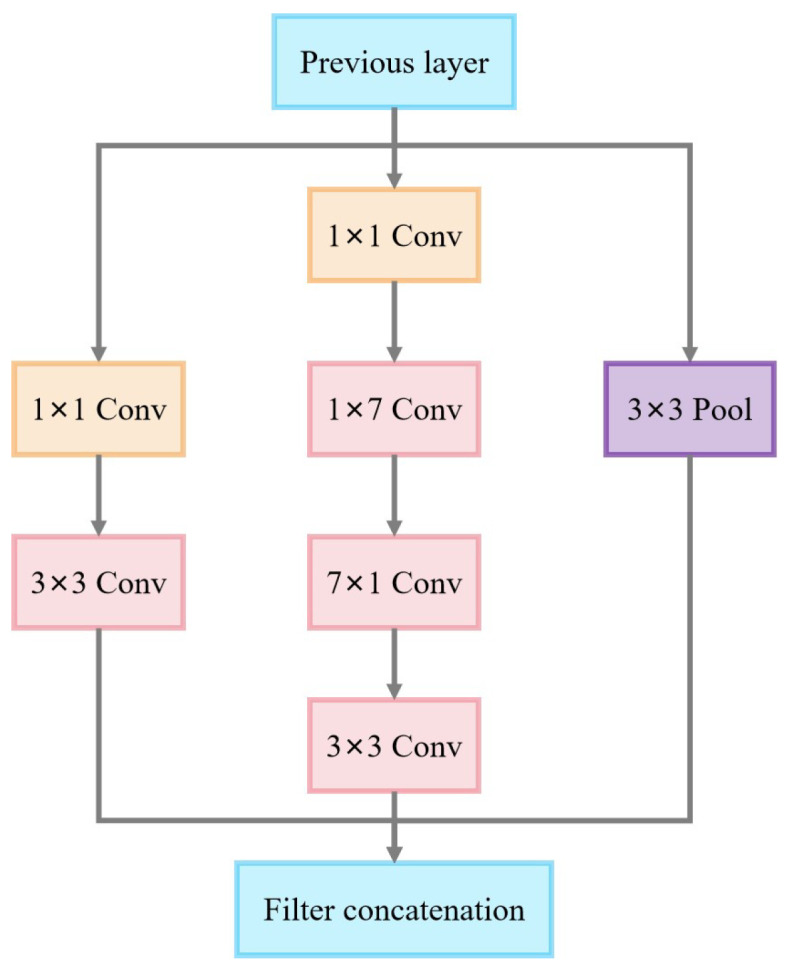
Inception v3-E module.

As shown in Figure 13, the Inception v3-E module employs a three-branch structure for feature extraction. The main branch performs basic feature extraction using 1 × 1 and 3 × 3 convolutions in series. In the middle branch, 1 × 1 convolution is used for dimensionality reduction, followed by 1 × 7 and 7 × 1 asymmetric convolution pairs to replace traditional large-size convolutions. Finally, 3 × 3 convolution is applied, which is a decomposition method that reduces computational costs. The third branch employs 3 × 3 pooling to retain the primary features.

#### 3.2.3. Efficient Linear Support Vector Machines

The core idea of SVM learning is to find the separating hyperplane that correctly divides the training dataset while maximizing the geometric margin. Assuming that the training dataset *D* is linearly separable, there are infinitely many hyperplanes (i.e., decision boundaries) for a linearly separable dataset, but the separating hyperplane with the largest geometric margin is unique.

(6) $$D=\{(x_{1},y_{1}),(x_{2},y_{2}),\ldots ,(x_{i},y_{i})\}$$

where $x_{i}\in R^{n}$, $y_{i}\in \{+1,-1\},i=1,2,\ldots ,N$, $x_{i}$ are the ith feature vectors and $y_{i}$ is the class labelling, which is a positive case when it is equal to +1 and a negative case when it is −1.

Taking the dataset *D* and the hyperplane ω⋅x+b=0, define the geometric interval of the hyperplane about the sample point $\left(x_{i},y_{i}\right)$ as follows:

(7) $$\gamma =y_{i}(\frac{w}{\left\|w\right\|}\cdot x_{i}+\frac{b}{\left\|w\right\|})$$

The minimum value of the geometric margin of the hyperplane with respect to all sample points is as follows:

(8) $$\gamma =\underset{i=1,2,\cdots ,N}{\min}\gamma_{i}$$

The SVM model for solving the maximum margin hyperplane problem can be expressed as the following constrained optimization problem:

(9) $$\underset{w,b}{\max}\;\gamma \;s.t.\;\;\;y_{i}(\frac{w}{\left\|w\right\|}\cdot x_{i}+\frac{b}{\left\|w\right\|})\geq \gamma ,i=1,2,\ldots ,N$$

where $\left\|w\right\|$, b are scalars.

Let $w=\frac{w}{\left\|w\right\|\gamma }$ and $b=\frac{b}{\left\|w\right\|\gamma }$. The original problem is transformed into the following quadratic programming problem:

(10) $$\min\frac{1}{2}{\left\|w\right\|}^{2}\;s.t.\begin{array}{cc} y_{i}(w\cdot x_{i}+b)\geq 1 & i=1,2,\cdots ,N \end{array}$$

The original objective function with constraints is transformed into the newly constructed Lagrangian objective function without constraints, yielding its Lagrangian dual problem as follows:

(11) $$L(w,b,\alpha )=\frac{1}{2}{\left\|w\right\|}^{2}-\sum_{i=1}^{N}\alpha_{i}(y_{i}(w\cdot x_{i}+b)-1)$$

where $\alpha_{i}$ is a Lagrange operator and $\alpha_{i}\geq 0$.

Let $\theta (w)=\underset{a_{i}\geq 0}{\max}L(w,b,\alpha )$, then the following two conditions are satisfied:

- When the sample points do not satisfy the constraints, i.e., they are outside the feasible solution region, $y_{i}(w\cdot x_{i}+b)<1$. At this point, $\alpha_{i}$ is set to infinity; then, θ(w) is also infinite.
- When the sample points satisfy the constraints, i.e., they are inside the feasible solution region, $y_{i}(w\cdot x_{i}+b)\geq 1$. At this point, θ(w) is the function itself.

Finding the greatness of $\underset{w,b}{\min}L(w,b,\alpha )$ over α yields the following dyadic problem:

(12) $$\underset{\alpha }{\max}\;\;\;-\frac{1}{2}\sum_{i=1}^{N}\sum_{j=1}^{N}\alpha_{i}\alpha_{j}y_{i}y_{j}(x_{i}\cdot x_{j})+\sum_{i=1}^{N}\alpha_{i}\;s.t.\left\{\begin{array}{c} \sum_{i=1}^{N}\alpha_{i}y_{i}=0\\ \alpha_{i}\geq 0,i=1,2,\ldots ,N \end{array}\right.$$

To avoid complex operations in high-dimensional feature space, this experiment uses a linear kernel function *K*, which satisfies the following:

(13) $$K(x_{i},x_{j})=(x_{i}\cdot x_{j})$$

Solving the problem yields $\alpha^{*}={\left[\alpha_{1}^{*},\alpha_{2}^{*},\ldots ,\alpha_{i}^{*}\right]}^{T}$. $w^{*}=\sum_{i=1}^{N}a_{i}^{*}y_{i}x_{i}$. Choose a positive component $\alpha_{j}^{*}$ of $\alpha^{*}$ and compute it as follows:

(14) $$b^{*}=y_{j}-\sum_{i=1}^{N}\alpha_{i}^{*}y_{i}(x_{i}\cdot x_{j})$$

Construct the following classification function:

(15) $$f(x)=sign(w^{*}\cdot x+b^{*})$$

## 4. Experimentation and Analysis

### 4.1. Experimental Procedure

#### 4.1.1. Parameter Calibration

The railway worker detection dataset has 3222 images, and after data enhancement, the sample size is 4048. Firstly, the dataset is divided into 14 scenes, and due to the large differences in the scene samples, it needs to be customised to divide each scene into 5 categories, to ensure that it can identify the features of the different categories in different scenes, in which category 0 represents the number of people in the scene as 0, category 1 represents the number of people in the scene as 1 person, category 2 category represents that the number of people in the scene has 2 people, category 3 represents that the number of people in the scene has 3 people, and category 4 means that the number of people in the scene is ≥4 people; the highest number of people in the dataset is only 8 people, and category including 4–8 people has fewer samples, which makes it difficult to use in training to identify the features, so the data are categorised as 4 categories, and the categories can be increased according to the actual situation when the sample size is large. The dataset in this study is divided using stratified sampling, specifically based on the 14 scenes in the dataset, according to different categories (some scenes lack categories), divided according to 60% for the training set, 25% for the testing set, and 15% for the validation set. For these scenarios, the division according to this ratio can ensure the reasonable distribution of each category in different scenarios, achieve the detection task in different scenarios, and avoid the model bias towards most categories. From the research objectives, the high generalisability model needs to accurately assess the performance; 25% of the test set is used to simulate real scenarios to assess the generalisation ability and 15% of the validation set monitors the performance during training to prevent overfitting and adjust the hyperparameters in time. The actual sample size of the division is shown in Table 2.

Table 3 presents the algorithm parameters for this experiment, where the image is first converted into a greyscale map before NLM processing.

#### 4.1.2. Results

**Figure 14:**
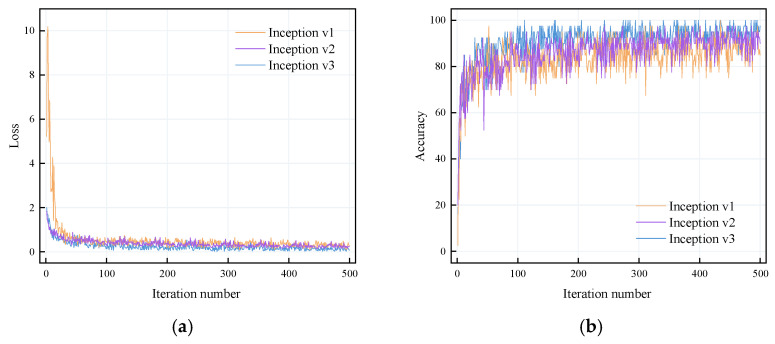
Iterative comparison experiment: (**a**) loss function and (**b**) training accuracy.

In this study, iterative comparative experiments were conducted among several variants of the Inception series models. As illustrated in Figure 14, Inception v3 demonstrated relatively faster convergence. Among these models, Inception v3 exhibited superior convergence speed compared to others, whereas Inception v3 outperformed Inception v1 regarding accuracy and the rate of parameter optimization.

**Figure 15:**
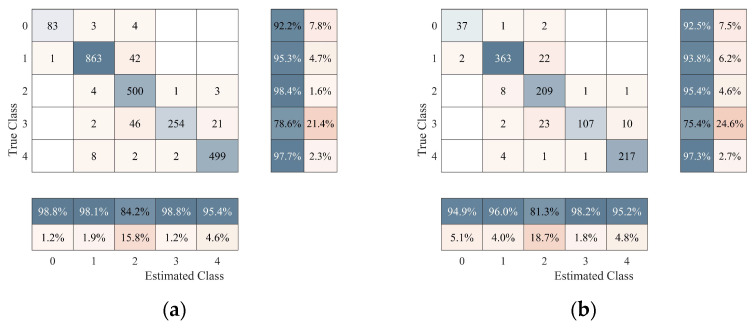
Confusion matrix: (**a**) training set and (**b**) test set.

Figure 15 illustrates the classification performance of the model on both the training and test sets. The confusion matrix for the training set shows high classification accuracy, particularly for categories 1 and 4, which perform well at 95.3% and 97.7%, respectively. However, category 3 has a relatively low accuracy of 78.6% and is more challenging to recognize. The results for the test set are similar to those of the training set, with a slight decrease in overall accuracy, particularly for category 3, where the accuracy drops to 75.4%. These results demonstrate that the model has good generalization ability for most categories but still requires further optimization for some categories.

As shown in Table 4, with 200 iterations, the ISVM algorithm improves accuracy over Inception v3 by 6.35% in the training set and 6.3% in the test set.

#### 4.1.3. Analysis of Differences in Classification

**Figure 16:**
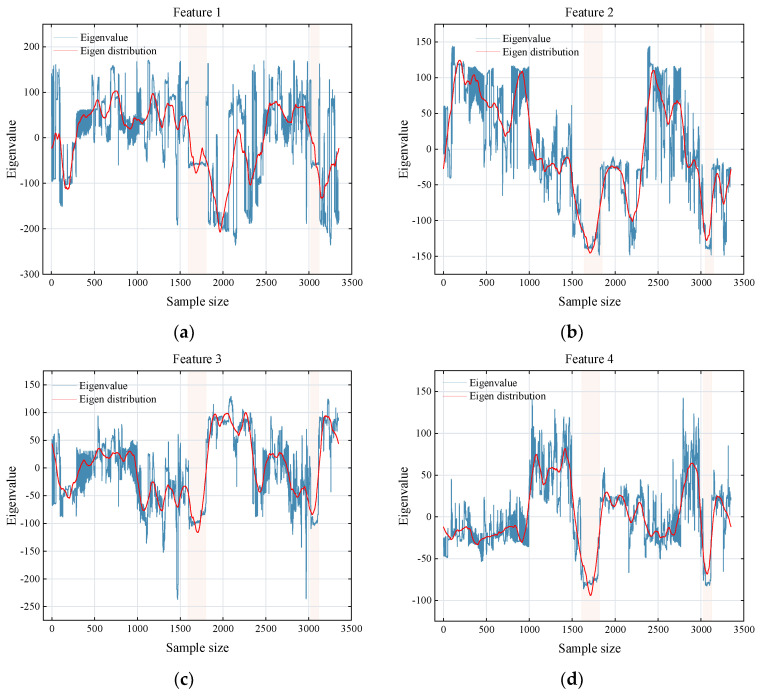

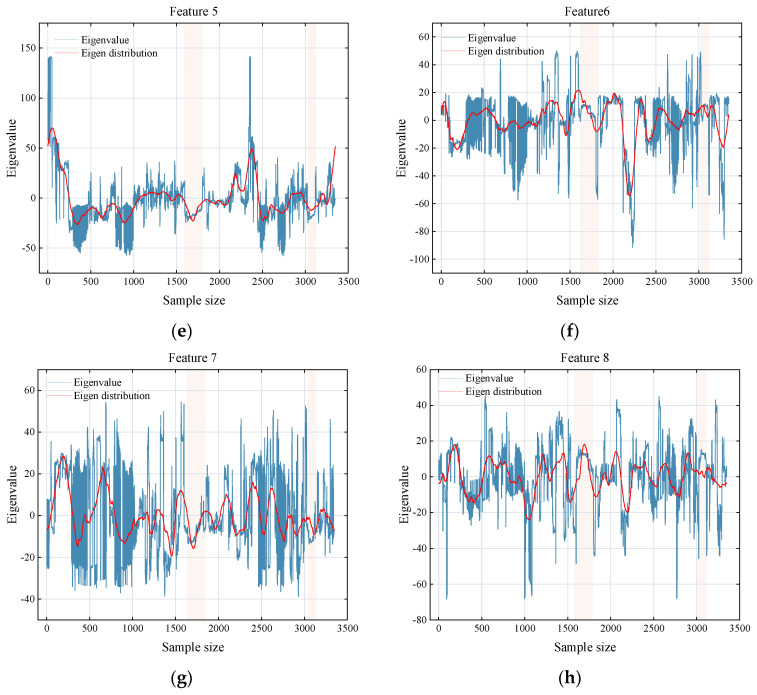
Feature mapping after dimensionality reduction. The image sample features (**a**–**h**) are extracted using Inception v3, and PCA is applied to select the features with a cumulative contribution rate of more than 95%, and 8 feature maps are obtained.

As shown in (a)–(h) of Figure 16, the image sample features are extracted using Inception v3, and PCA is applied to select the features with a cumulative contribution rate of more than 95%, and 8 feature maps are obtained as shown in Figure 16. It is found that the reason for the higher error rate of category 3 is: ① the difference in scene changes is small, resulting in low differentiation of features learned after feature extraction; ② the 8 features of category 3 have little change and are difficult to recognize; ③ the data sample size is small. The features of other categories have higher differentiation, so the prediction performance is relatively good.

**Figure 17:**
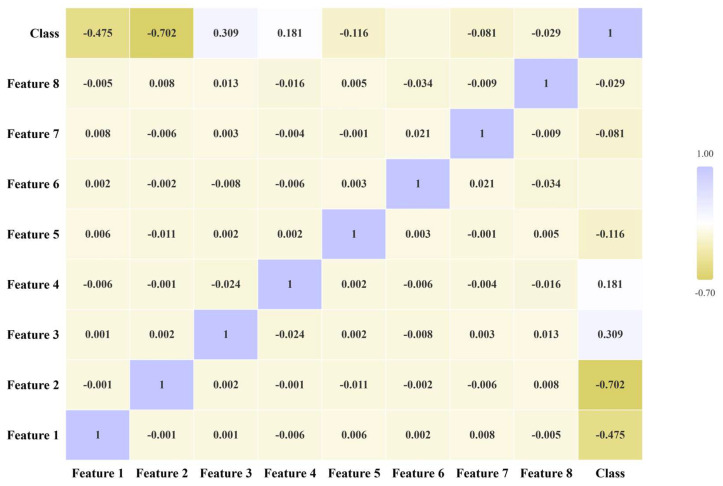
Heat map for Pearson correlation analysis.

The correlation experiments revealed that, as shown in Figure 17, the correlation between the features extracted by Inception v3 is extremely low, indicating that the features are essentially independent of each other and that there is no multicollinearity problem. Therefore, a linear kernel function is more suitable for classification.

### 4.2. Experiments and Discussions

#### Performance Evaluation

In this study, all experiments were conducted on computers with the following configuration: 13th Gen Intel Core i7-13650HX (20 cores, approx. 2.6 GHz), 16 GB RAM, Dell G16 7630.

This study begins with feature extraction from the AvgPool layer using the Inception v3 structure. The performance of each classifier was systematically evaluated through 50 independent experiments by feeding the feature vectors into 25 classifiers for the classification task (shown in Table 5).

The experimental results show that the efficient linear SVM and logistic regression exhibit excellent overall performance with short training times, fast prediction speeds, and 92.3% accuracy. The bagging tree classifier has the fastest prediction speed (2542.56 obs/s) and 91.8% accuracy, although its training time is relatively long (95.14 s). Stability analysis shows that some classifiers exhibit good robustness and do not overfit when features change.

As shown in Table 6, after dimensionality reduction using PCA to select features with an accumulated contribution rate greater than 95%, the accuracy of some classifiers has been improved to a certain extent, but it requires a certain amount of training time.

In this experiment, the AUC, TPR, and FPR metrics are used to evaluate the classifier performance of models.

TPR is the sensitivity or recall rate, which represents the proportion of actual positive samples correctly predicted as positive examples, and is calculated as follows:

(16) $$TPR=\frac{TP}{TP+FN}$$

where TP (True Positive) refers to the number of samples correctly predicted as positive, and FN (False Negative) refers to the number of samples incorrectly predicted as negative.

The FPR, also known as the False Positive Rate, represents the proportion of actual negative samples incorrectly predicted as positive, and is calculated as follows:

(17) $$FPR=\frac{FP}{FP+TN}$$

where FP (False Positive) refers to the number of samples incorrectly predicted as positive, and TN (True Negative) refers to the number of samples correctly predicted as negative.

AUC is the area under the ROC curve, representing the likelihood that the classifier will predict a positive sample as positive with a higher probability than a negative sample by randomly selecting a pair from all positive and negative sample pairs. The formula is as follows:

(18) $$AUC=\int_{0}^{1}TPR(FPR)dFPR$$

**Figure 18:**
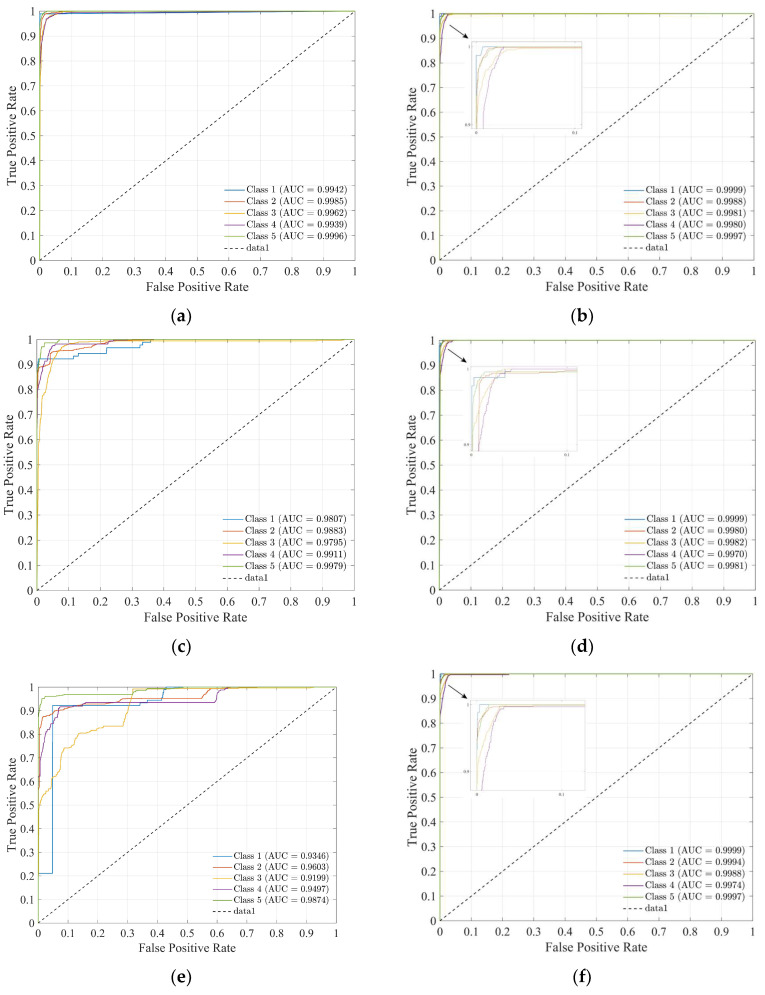
ROC curves: (**a**,**b**) efficient linear SVM, (**c**,**d**) efficient logistic regression, and (**e**,**f**) bagging tree.

Figure 18 shows the ROC curves for each category of the three models (validation). The closer the curve is to the upper left corner, the better the classifier’s performance. (a), (c), and (e) represent the models trained without dimensionality reduction, while (b), (d), and (f) represent the models trained with PCA-dimensionality-reduced features. The experimental results show that the efficient linear SVM has strong feature recognition ability, and the AUC can approach one in both cases. Introducing PCA dimensionality reduction significantly improves the AUC of efficient logistic regression and the bagging tree.

## 5. Discussion

**Figure 19:**
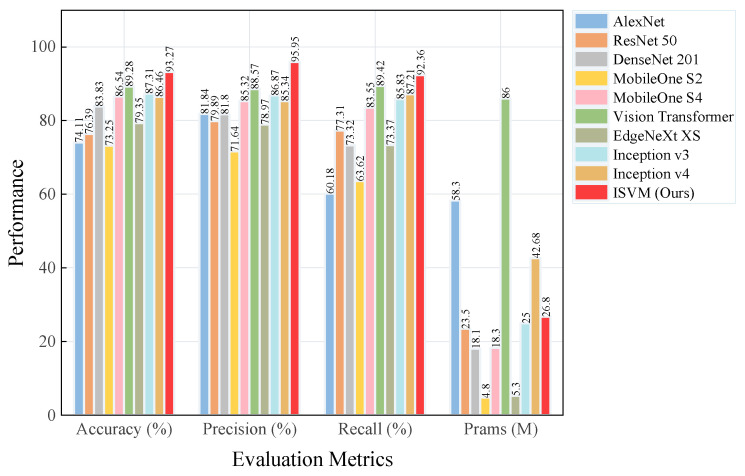
Model evaluation.

In the comparative experiments, we selected representative models covering different architectures and complexities to comprehensively verify the effectiveness and generalisation ability of the method. To ensure the fairness of the experiment, all models have an input size of 224 × 224 pixels, 200 stops per round, a batch size of 32, and an initial learning rate of 0.0001. As shown in Figure 19, the main evaluation metrics are accuracy, precision, recall, and the number of parameters, which are used to evaluate the performance and learning ability of the algorithm for short-term prediction.

As shown in Table 7, The experimental results show that the proposed ISVM model is significantly better than the comparison models in terms of accuracy, precision, recall, and F1 score. The ISVM model improves the average values of the comparison models by 11.43%, 13.70%, 15.27%, and 17.12% in terms of accuracy, precision, recall, and F1 score, respectively. The vision transformer performs well in terms of accuracy, but its parameters are as high as 86 M, which is less computationally efficient. Lightweight models such as EdgeNeXt-XS and MobileOne-S2 have relatively poor prediction results. ISVM achieves a balance of performance and efficiency with 26.80 M parameters. 

## 6. Conclusions

This study designed an ISVM method combined with image enhancement technology to detect railway construction safety. The following conclusions were drawn:

(1) By combining deep learning and traditional machine learning methods, the ISVM algorithm improves the accuracy of the Inception v3 model by 6.3%, and the accuracy, precision, recall, and F1 score indicators are improved by 11.43%, 13.70%, 15.27%, and 17.12%, respectively, compared to the average of the comparison models. The prediction accuracy is improved in short-term prediction.
(2) This experiment uses principal component analysis to reduce the dimensionality of the features of the AvgPool layer of the Inception v3 network. The experimental results show that the features are basically independent of each other, and there is no obvious problem of multiple collinearity. However, the recognition accuracy of some categories is relatively low, which is caused by the uneven distribution of the sample size of various categories in the scene and the low degree of feature differentiation.
(3) This experiment tests the performance of 25 classifiers on the validation set. The results show that introducing PCA dimensionality reduction improves the prediction speed of most classifiers, but it also increases training time. Among them, the efficient linear SVM shows a 1.9% improvement in accuracy and a 2405.49 obs/s improvement in prediction speed. Notably, the efficient linear SVM, with or without dimensionality reduction, demonstrates strong adaptability, particularly when handling high-dimensional features, and exhibits better robustness and generalization ability. Additionally, the bagging tree classifier performs particularly well in terms of prediction speed and is suitable for large-scale real-time detection tasks. These results suggest that selecting an appropriate classifier is crucial for enhancing the overall performance of the detection system.

In terms of future research directions, the introduction of multimodal data can be considered. A single visual image has limitations when faced with complex conditions such as different lighting and occlusions. Combining information such as infrared images and depth images can effectively supplement its deficiencies. Infrared images can highlight the thermal radiation characteristics of the human body at night or in dark environments, and clearly show the position of construction personnel even in poor lighting conditions. Depth images can provide depth information about the scene, helping models to better understand the spatial position relationships of objects, and more accurately determine the status and location of personnel in the event of occlusion, thereby significantly improving detection performance. Exploring new neural network structures and optimising existing models with the lightweight model design concept are important future tasks. New neural network structures may have more efficient feature extraction and processing capabilities, which can improve computational efficiency while ensuring detection accuracy. The lightweight model design concept focuses on reducing model parameters and computational complexity and reducing dependence on hardware resources. By combining the two, existing models can be optimised, model parameters and computational complexity can be reduced, and models can run quickly on hardware devices with limited resources, enabling faster and more accurate detection of railway construction personnel and effectively meeting the dual needs of detection efficiency and accuracy in practical engineering applications.

## Figures and Tables

**Table 1:** Summary of literature.

Author	Algorithm Used	Algorithm Model	Application Domain	Model Fusion	
				Yes	No
Ying [17]	Convolutional Neural Networks with Support Vector Machines	Deep learning	Construction safety	√	
Liu [18]	Genetic Algorithm Optimization of BP Neural Networks	Deep learning	Construction safety	√	
Yang [19]	Background Difference Method	Information fusion model	Construction safety	√	
Nogueira K [30]	Dynamically Expanded Convolutional Networks	Deep learning	Rail monitoring		√
Cao [20]	Lightweight Neural Networks	Deep learning	Construction safety		√
Zhang [28]	YOLOv5 Neural Network	Target detection algorithms	Construction safety	√	
Kapoor R [32]	Deep Classifier Network	Deep learning	Rail monitoring	√	
Cheng [35]	NLRA	Pattern recognition	Rail monitoring	√	
Xu [23]	Convolutional Recurrent Neural Network	Deep learning	Equipment Monitoring	√	
Aydin [26]	LCNN	Machine learning	Equipment Monitoring	√	
Yue [36]	YOLOP	Target detection algorithms	Rail monitoring		√
Gibert [25]	Artificial Neural Network	Deep learning	Equipment Monitoring	√	
Tan [27]	Faster R-CNN	Target detection algorithms	Equipment Monitoring	√	
Min [41]	DR-VAE	Unsupervised learning	Rail monitoring	√	
Kim [38]	Fully Convolutional Network	Deep learning	Rail monitoring		√
Rampriya [44]	Deep Residual U-Net	Machine learning	Rail monitoring	√	
Wei [29]	PDDNet	Deep learning	Equipment Monitoring	√	
Zhuang [42]	A Data-Driven Approach	Deep learning	Rail monitoring	√	
Guo [37]	Finite Element Analysis (FEA)	Finite element simulation model	Rail monitoring	√	

**Table 2:** Dataset classification.

Category	Training Set	Test Set	Validation Set	Total
0	90	40	28	158
1	906	387	155	1448
2	508	219	265	992
3	323	142	187	652
4	511	223	64	798
Total	2338	1011	699	4048

**Table 3:** Calibration of algorithm parameters.

Algorithm	Parameter Meaning	Setting Value
NLM	Image resolution	1920 × 1080
	Convert image type	Grey
	Search window size	15
	Image block size	9
	Smoothing parameters	21
Histogram Equalization	Number of bins in the histogram	256
	Contrast enhancement factor	0
	Brightness enhancement factor	0
	gamma correction factor	1
Inception v3	Input size	299 × 299 × 3
	Learning rate	0.001
	Maximum number of iterations	500
	Validation frequency	50
	Batch size	32
	Dropout ratio	0.2
	Weight decay	0.0001
	Optimizer	SGD
	Activation function	ReLU
Efficient Linear SVM	K-fold cross validation	5
	Test set ratio	0.3
	Regularization parameters	10
	Penalty factor	10
	Tolerance	0.001
	Maximum number of iterations	1000
	Loss function	Modified Huber loss
	Kernel function	Linear kernel function

**Table 4:** Accuracy.

Algorithm	Training Set	Test Set	Training Time (s)
Inception v3	87.21%	87.17%	184
Inception v2	85.73%	84.82%	169
Inception v1	84.59%	84.48%	153
ISVM	93.56%	93.47%	196

**Table 5:** Predictive performance of different classifiers (without dimensionality reduction).

Model Type	Presuppose	Accuracy (Verification)	Total Cost (Validation)	Predicted Speed (obs/s)	Training Time (Seconds)
Efficient Logistic Regression	Efficient Logistic Regression	0.923 ± 0.02	60	1882.77	14.94
Efficient Linear SVM	Efficient Linear SVM	0.923 ± 0.03	65	2275.27	11.36
Plain Bayes	Kernel Plain Bayes	0.851 ± 0.04	178	31.16	309.71
Tree	Fine Tree	0.905 ± 0.02	90	1669.01	25.88
	Medium Tree	0.905 ± 0.02	90	1536	22.09
	Coarse Tree	0.915 ± 0.03	73	1669.66	20.59
SVM	Linear SVM	0.910 ± 0.03	65	1449.27	38.45
	Quadratic SVM	0.912 ± 0.01	62	1230.5	36.99
	Cubic SVM	0.919 ± 0.01	67	1199.04	36.21
	Fine Gaussian SVM	0.916 ± 0.03	71	802.68	34.93
	Medium Gaussian SVM	0.918 ± 0.03	69	1129.29	34.27
	Rough Gaussian SVM	0.876 ± 0.03	137	964.36	32.89
KNN	Fine KNN	0.904 ± 0.03	92	657.78	31.79
	Medium KNN	0.898 ± 0.02	102	657.33	30.55
	Coarse KNN	0.791 ± 0.05	277	754.07	29.85
	Cosine KNN	0.898 ± 0.03	102	582.59	28.62
	Cubic KNN	0.905 ± 0.03	90	183.64	85.69
	Weighted KNN	0.889 ± 0.03	99	1109.51	27.56
Integration	Lifting Trees	0.907 ± 0.03	87	2034.68	86.82
	Bagging tree	0.918 ± 0.03	78	2542.56	95.14
	Subspace discriminant	0.917 ± 0.02	71	393.49	63.4
	Subspace KNN	0.909 ± 0.03	83	167.54	70.2
	RUSBoosted tree	0.909 ± 0.03	82	1297.62	54.28
Kernel	SVM Kernel	0.902 ± 0.01	62	607.04	207.76
	logistic regression kernel	0.919 ± 0.03	66	297.83	129.09

**Table 6:** Predictive performance of different classifiers (after dimensionality reduction).

Model Type	Presuppose	Accuracy (Verification)	Total Cost (Validation)	Predicted Speed (obs/s)	Training Time (Seconds)
Efficient Logistic Regression	Efficient Logistic Regression	0.942 ± 0.02	62	4819.49	622.89
Efficient Linear SVM	Efficient Linear SVM	0.944 ± 0.03	59	4680.76	603.23
Plain Bayes	Kernel Plain Bayes	0.922 ± 0.01	79	854.23	621.10
Tree	Fine Tree	0.929 ± 0.01	84	1947.13	18.20
	Medium Tree	0.931 ± 0.02	81	1870.18	627.04
	Coarse Tree	0.862 ± 0.05	193	1857.38	625.21
SVM	Linear SVM	0.935 ± 0.01	73	1731.71	620.14
	Quadratic SVM	0.939 ± 0.02	68	1440.99	619.46
	Cubic SVM	0.911 ± 0.03	64	1478.08	618.66
	Fine Gaussian SVM	0.938 ± 0.01	69	1704.47	617.73
	Medium Gaussian SVM	0.918 ± 0.01	101	1501.79	616.86
	Rough Gaussian SVM	0.868 ± 0.05	1002	1560.30	615.82
KNN	Fine KNN	0.931 ± 0.01	80	2077.37	619.01
	Medium KNN	0.935 ± 0.01	73	1945.83	618.41
	Coarse KNN	0.893 ± 0.03	142	1644.69	617.92
	Cosine KNN	0.939 ± 0.02	67	1918.58	617.27
	Cubic KNN	0.935 ± 0.02	74	3323.83	616.66
	Weighted KNN	0.932 ± 0.03	78	3952.22	615.85
Integration	Lifting Trees	0.915 ± 0.02	74	2085.90	615.13
	Bagging Tree	0.940 ± 0.01	82	5038.28	613.99
	Subspace Discriminant	0.933 ± 0.02	68	1001.08	631.19
	Subspace KNN	0.934 ± 0.02	75	766.98	18.01
	RUSBoosted Tree	0.939 ± 0.03	68	1408.36	17.35
Kernel	SVM Kernel	0.935 ± 0.03	73	2483.67	23.54
	Logistic Regression Kernel	0.922 ± 0.03	63	2021.97	19.30

**Table 7:** Comparison of experimental results.

Model	Accuracy Rate (%)	Precision Rate (%)	Recall Rate (%)	F1 Score (%)	Param (M)
AlexNet	74.11	81.84	60.18	56.24	58.30
ResNet-50	76.39	79.89	77.31	77.39	23.50
DenseNet-201	83.83	81.80	73.32	73.39	18.10
MobileOne-S2	73.25	71.64	63.62	62.64	4.80
MobileOne-S4	86.54	85.32	83.55	83.51	18.30
Vision Transformer	89.28	88.57	89.42	88.14	86.00
EdgeNeXt-XS	79.35	78.97	73.37	71.25	5.30
Inception v3	87.31	86.87	85.83	85.81	25.00
Inception v4	86.46	85.34	87.21	87.18	42.68
ISVM (Ours)	93.27	95.95	92.36	93.30	26.80

## Data Availability

The datasets presented in this article are not readily available because the data are part of an ongoing study. Requests to access the datasets should be directed to guguobin@unn.edu.cn.
